# The hemoglobin level impact on arterial oxygen saturation during venous-venous-extracorporeal membrane oxygenation support of acute respiratory distress syndrome patients: a mathematical marginal approach

**DOI:** 10.5935/0103-507X.20220465-en

**Published:** 2022

**Authors:** Luisa Tajra Carvalho, Pedro Vitale Mendes, Bruno Adler Maccagnan Pinheiro Besen, Marcelo Park

**Affiliations:** 1 Emergency Medicine Discipline, Hospital das Clínicas, Faculdade de Medicina, Universidade de São Paulo - São Paulo (SP), Brazil.

## TO THE EDITOR

Hemoglobin (Hb) levels in the range of 7 - 14g/dL have been targeted in extracorporeal membrane oxygenation (ECMO)-supported acute respiratory distress syndrome (ARDS) patients. There is an association between low Hb levels and prolonged duration of mechanical ventilation and bleeding episodes. In contrast, higher Hb levels are associated with lower ECMO blood flow, increased hemolysis, and increased costs. Current transfusion strategies are mostly based on individual judgment, derived mainly from oxygen delivery (DO_2_) /consumption rationale (VO_2_).^(1)^ High volume ECMO centers are used to more restrictive Hb strategies, although there is no consensus on a definitive transfusion approach.^(2)^ Conversely, some experienced centers use higher Hb thresholds for transfusion and accept oxygen arterial saturation (SatO_2_) as low as 70% with excellent clinical outcomes.^(3)^

Critical illnesses are related to cellular dysfunction due to reduced DO_2_ to tissues. Oxygen delivery depends on cardiac output (CO), Hb level, oxygen arterial partial pressure (PaO_2_), and SatO_2_ as in equation 1.^(4)^


Equation 1 $${DO}_{2}=CO\times \left(\left[Hb\times {SatO}_{2}\times 1.36\right]+\left[0.0031\times {PaO}_{2}\right]\right)$$

The physiological role of SatO_2_ on DO_2_ is crucial, with the oxygen bound to hemoglobin accountable for the majority of the blood’s oxygen content. Additionally, because the dissolved O_2_ content in plasma is negligible in normobaric conditions, it can be excluded from calculation of DO_2_.^(4)^ As the main goal of venous-venous (VV)-ECMO is to provide adequate DO_2_, VV-ECMO oxygenation settings are mostly based on SatO_2_.

While the impact of Hb levels on DO_2_ in ECMO-supported patients has been previously modeled, the effect size of Hb levels on SatO_2_ remains still unclear.^(5)^ We used a previously described mathematical marginal multicompartmental model of systemic SatO_2_ during femoro-jugular VV-ECMO support.^(6)^ This model accounts for recirculation proportional to ECMO blood flow and systemic, native lung and artificial lung compartments. To assess the effect of Hb level on systemic SatO_2_, we contrasted different scenarios related to patient and ECMO variables, such as systemic VO_2_ rates, ECMO blood flow and CO, to highlight the dynamic care required by such patients. The behavior of dual lumen bicaval and femoro-femoro (venous-venous) configurations are probably similar but with a slightly increased recirculation.

The R free source software was used for the mathematical modeling and graphical buildings. The script of the model is freely accessible on the website.

**Figure 1:**
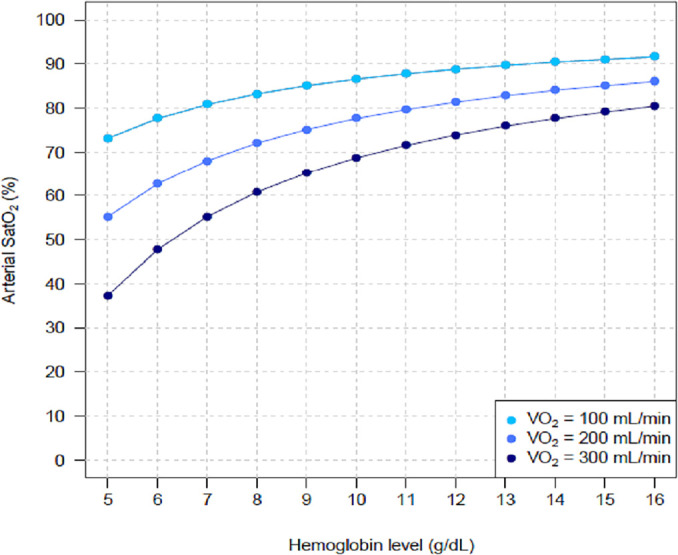
Arterial oxygen saturation behavior with progressively higher hemoglobin levels under three different systemic oxygen consumptions.

**Figure 2:**
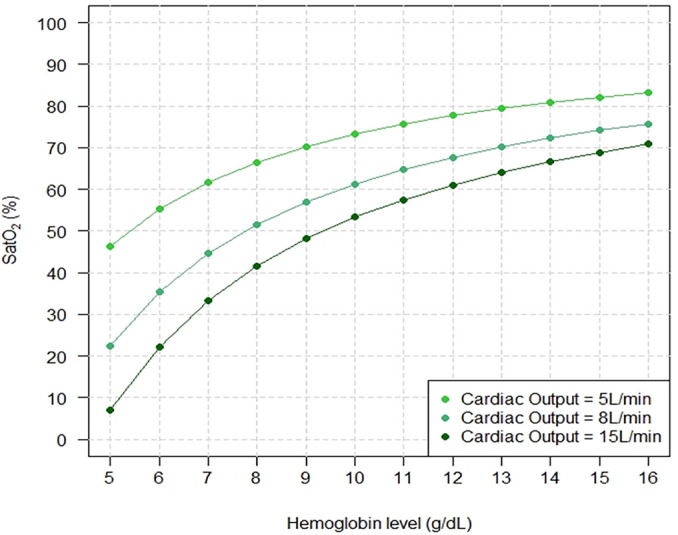
Arterial oxygen saturation behavior with progressively higher hemoglobin levels under three different cardiac outputs.

**Figure 3:**
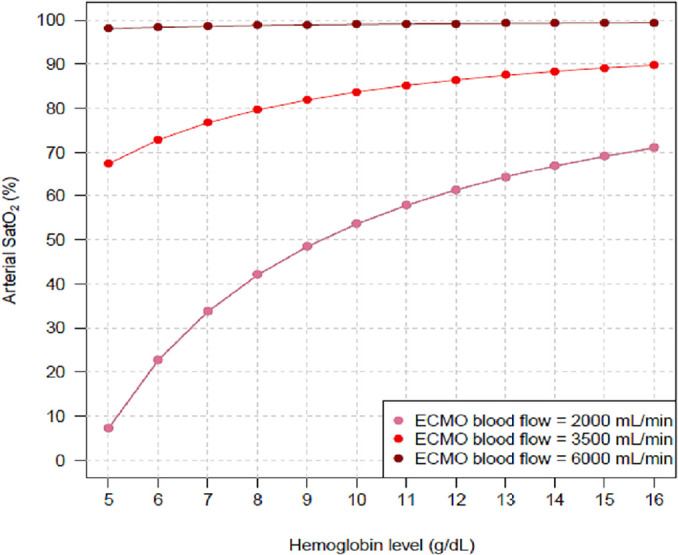
Arterial oxygen saturation behavior with progressively higher hemoglobin levels under three different blood flows with extracorporeal membrane oxygenation.

**Figure 4:**
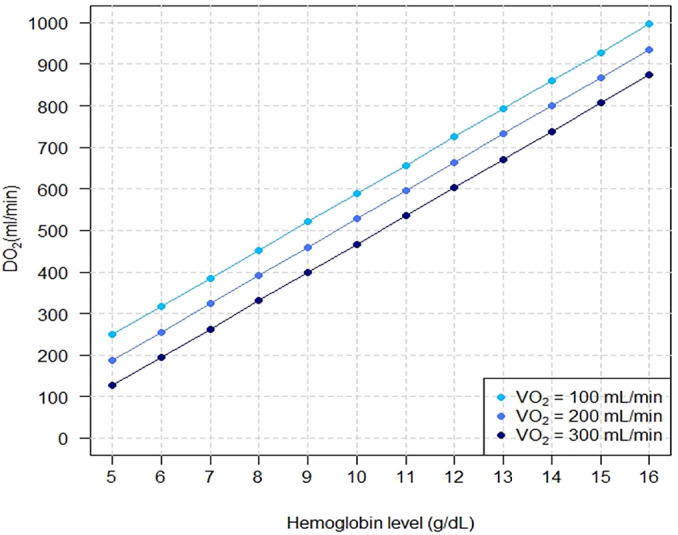
Systemic oxygen delivery with progressively higher hemoglobin levels under three different systemic oxygen consumptions.

Figure 1 shows the results of Hb level influence on SatO_2_ under three different VO_2_ levels. Figure 2 shows the same impact under fixed VO_2_ and different COs, while figure 3 shows the same impact under fixed VO_2_ and CO but with different ECMO blood flows. Figure 4 shows the linear relationship between Hb levels and DO_2_.

The controlled variables were as follows: cardiac output = 5L/minute; extracorporeal membrane oxygenation blood flow = 3.5L/minute; partial pressure of carbon dioxide = 40mmHg; fraction of inspired oxygen with mechanical ventilator = 0.3; pulmonary shunt fraction = 0.9; and fraction of inspired oxygen with extracorporeal membrane oxygenation = 1.

SatO_2_ - systemic arterial oxygen saturation; VO_2_ - oxygen consumption.

The controlled variables were as follows: oxygen consumption = 300mL/minute; extracorporeal membrane oxygenation blood flow = 3.5L/minute; partial pressure of carbon dioxide = 40mmHg; fraction of inspired oxygen with mechanical ventilator = 0.3; pulmonary shunt fraction = 0.99; fraction of inspired oxygen with extracorporeal membrane oxygenation = 1.

SatO_2_ - systemic arterial oxygen saturation.

The controlled variables were as follows: oxygen consumption = 300mL/minute; cardiac output = 5L/min; partial pressure of carbon dioxide = 40mmHg; fraction of inspired oxygen with mechanical ventilator = 0.3; pulmonary shunt fraction = 0.9; fraction of inspired oxygen with extracorporeal membrane oxygenation = 1.

SatO_2_ - systemic arterial oxygen saturation; ECMO - extracorporeal membrane oxygenation.

The controlled variables were cardiac output = 5L/minute; blood flow with extracorporeal membrane oxygenation = 3.5L/minute; partial pressure of carbon dioxide = 40mmHg; fraction of inspired oxygen with mechanical ventilator = 0.3; pulmonary shunt fraction = 0.9; fraction of inspired oxygen with extracorporeal membrane oxygenation = 1; systemic oxygen delivery = cardiac output x Hb x SO_2_ x 1.36.

DO_2_ - systemic oxygen delivery; VO_2_ - oxygen consumption.

The results of these mathematical marginal simulations were compatible with increased SatO_2_ and DO_2_ when the Hb levels were higher. Other bedside physiological variables interacted with the relationship between Hb level and SatO_2_; hence, our findings reflect that for a fixed Hb level, a higher VO_2_, higher CO, and lower ECMO blood flow were associated with more severe hypoxemia.

The mechanism of such Hb impact on SatO_2_ is a matter of oxygen content. For the same VO_2_, CO, and ECMO blood flow, a higher Hb level provides a higher arterial oxygen content; therefore, the residual venous oxygen content will also be increased, resulting in a higher venous oxygen saturation and consequently a higher SatO_2_ after oxygenation through the native and artificial lungs.

The reported relationships are not intended to have a predictive role in clinical circumstances, since the model was constructed to reflect associations between the studied variables in a hypothetical steady state. Despite these limitations, our findings reflect important physiological concepts that can be incorporated into the rationale of managing severely hypoxemic patients on VV-ECMO support.

Among patients undergoing ECMO support, extremely hypoxemic circumstances are not an uncommon scenario, and intensivists may need to accept very low SatO_2_ levels. In such cases, higher Hb thresholds could be used to allow adequacy between VO_2_ and DO_2_. Additionally, our mathematical model can improve the understanding of the reasoning behind findings of very low SatO_2_ and satisfactory clinical outcomes in clinical practice.^(3)^ However, it remains fundamental to emphasize the possible deleterious effects of severe hypoxemia with the installation of pulmonary hypertension and right ventricular dysfunction, in addition to long-term cognitive effects.

In conclusion, higher levels of Hb are associated with increased DO_2_ and SatO_2_. This association is modulated, at least, by the cardiac output , systemic VO_2_, and ECMO blood flow.
